# Supplementary material for: TNF-α in Combination with Palmitate Enhances IL-8 Production via The MyD88- Independent TLR4 Signaling Pathway: Potential Relevance to Metabolic Inflammation
Source: Int J Mol Sci. 2019 Aug 23;20(17):4112. doi: 10.3390/ijms20174112 (PMC6747275; doi:10.3390/ijms20174112)
Supplement: Supplementary file 1 [file ijms-20-04112-s001.pdf]

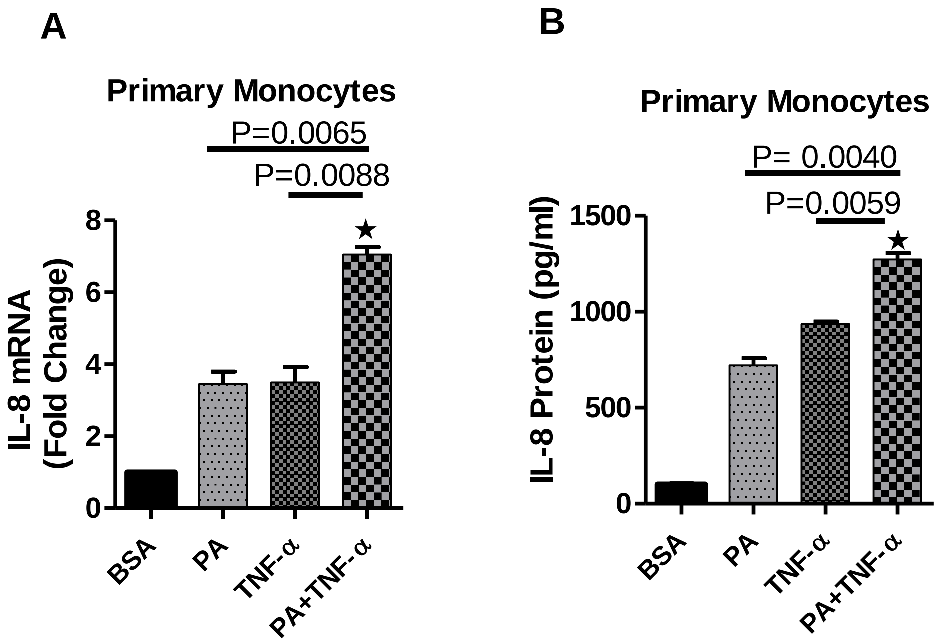

Supplemental Figure 1. Primary monocytes were isolated from PBMCs of healthy volunteers as described previously [38]. Monocytes were incubated with palmitate (15μM) and/or TNF-*α* (100pg) for 24 h. IL-8 mRNA (A) and secreted protein (B) were determined by real-time RT-PCR and ELISA. The results obtained from three replicates of each experiment are shown. Data are expressed as mean ± SEM.
